# Disentangling the impact of environmental and phylogenetic constraints on prokaryotic within-species diversity

**DOI:** 10.1038/s41396-020-0600-z

**Published:** 2020-02-11

**Authors:** Oleksandr M. Maistrenko, Daniel R. Mende, Mechthild Luetge, Falk Hildebrand, Thomas S. B. Schmidt, Simone S. Li, João F. Matias Rodrigues, Christian von Mering, Luis Pedro Coelho, Jaime Huerta-Cepas, Shinichi Sunagawa, Peer Bork

**Affiliations:** 1 European Molecular Biology Laboratory, Structural and Computational Biology Unit, 69117 Heidelberg, Germany; 2 Department of Molecular Life Sciences and Swiss Institute of Bioinformatics, University of Zurich, CH-8057 Zurich, Switzerland; 3 Max Delbrück Centre for Molecular Medicine, Berlin, Germany; 4 Molecular Medicine Partnership Unit, University of Heidelberg and European Molecular Biology Laboratory, Heidelberg, Germany; 5 Department of Bioinformatics, Biocenter, University of Würzburg, Würzburg, Germany; 6 Present Address: Laboratory of Applied Evolutionary Biology, Department of Medical Microbiology, Academic Medical Centre, University of Amsterdam, Amsterdam, 1105 AZ The Netherlands; 7 Present Address: Institute of Immunobiology, Kantonsspital St. Gallen, 9007 St. Gallen, Switzerland; 8 Present Address: Gut Microbes and Health, Quadram Institute Bioscience, Norwich, Norfolk UK; 9 Present Address: Digital Biology, Earlham Institute, Norwich, Norfolk UK; 10 Present Address: Novo Nordisk Foundation Center for Biosustainability, Technical University of Denmark, 2800 Kongens Lyngby, Denmark; 11 Present Address: Institute of Science and Technology for Brain-Inspired Intelligence, Fudan University, Shanghai, 200433 China; 12 Present Address: Centro de Biotecnología y Genómica de Plantas, Universidad Politécnica de Madrid (UPM) - Instituto Nacional de Investigación y Tecnología Agraria y Alimentaria (INIA), Madrid, Spain; 13 Present Address: Department of Biology and Swiss Institute of Bioinformatics, ETH Zürich, Vladimir-Prelog-Weg 4, 8093 Zürich, Switzerland

**Keywords:** Microbial ecology, Microbiology, Evolution

## Abstract

Microbial organisms inhabit virtually all environments and encompass a vast biological diversity. The pangenome concept aims to facilitate an understanding of diversity within defined phylogenetic groups. Hence, pangenomes are increasingly used to characterize the strain diversity of prokaryotic species. To understand the interdependence of pangenome features (such as the number of core and accessory genes) and to study the impact of environmental and phylogenetic constraints on the evolution of conspecific strains, we computed pangenomes for 155 phylogenetically diverse species (from ten phyla) using 7,000 high-quality genomes to each of which the respective habitats were assigned. Species habitat ubiquity was associated with several pangenome features. In particular, core-genome size was more important for ubiquity than accessory genome size. In general, environmental preferences had a stronger impact on pangenome evolution than phylogenetic inertia. Environmental preferences explained up to 49% of the variance for pangenome features, compared with 18% by phylogenetic inertia. This observation was robust when the dataset was extended to 10,100 species (59 phyla). The importance of environmental preferences was further accentuated by convergent evolution of pangenome features in a given habitat type across different phylogenetic clades. For example, the soil environment promotes expansion of pangenome size, while host-associated habitats lead to its reduction. Taken together, we explored the global principles of pangenome evolution, quantified the influence of habitat, and phylogenetic inertia on the evolution of pangenomes and identified criteria governing species ubiquity and habitat specificity.

## Introduction

Prokaryotic species vary ~100-fold in genome size and gene content [1]. The gene content of bacterial and archaeal genomes is mainly shaped by gene duplication, neo-/sub-functionalization, and losses. Other sources of functional innovation include the de novo emergence of genes and horizontal transfer, all leading to a vast prokaryotic genomic diversity [2–4]. In order to characterize strain diversity within a species, pangenome analyses have proven useful [5]. The pangenome is the non-redundant set of all genes (gene clusters or homologous groups) found in all genomes of a taxon [6, 7]. A species pangenome contains core genes (that are present in almost all isolates) and accessory genes, which can be further subdivided based on their prevalence. Each newly sequenced genome of a conspecific strain can contribute anywhere between 0 and more than 300 new genes to the pangenome of a species [8]. This potentially infinite addition of new genes due to horizontal gene transfer and other mechanisms means that the accessory gene repertoire of a species can theoretically increase with no emerging upper boundary, making pangenomes appear *open* [6, 9].

The pangenome of a given species is potentially shaped by its respective habitat(s) (via selection and drift) and phylogeny (inherited gene content after speciation). For example, previous studies have observed a relationship between habitat and genome size (as a proxy for gene content): free-living soil bacteria tend to have the largest described genomes [10, 11] while marine free-living and intracellular symbionts harbor the smallest ones [12–15]. Obligate symbiotic species tend to have small pangenomes—almost equal to the genome size, while soil-associated and some highly abundant free-living marine bacteria tend to have the largest pangenomes [16]. However, it is not well understood which aspects of a species’ pangenome are influenced by environmental factors and phylogenetic inertia. The overall architecture of a pangenome can be described from various angles, using the established quantitative measures of individual pangenome features, such as pan/core-genome sizes, genome fluidity, and average nucleotide identity/diversity (see Supplementary Table 1 for definitions of all metrics used in the present study). Many pangenome features describe the size of certain categories of genes, while others focus on a description of within-species diversity.

**Fig. 1:**
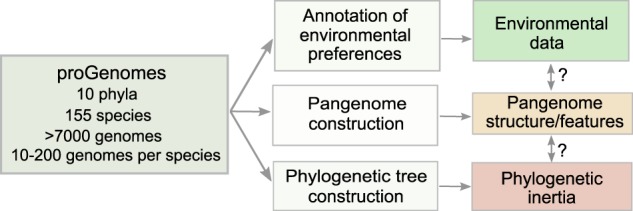
Study design. We used the proGenomes database version 1 [32] of high-quality genomes to compute pangenomes (using the Roary pipeline) and pangenome features. Species were assigned to their preferred habitats using three databases: PATRIC, Microbe Atlas Project, and Global Microbial Gene Catalog (see Methods). As many pangenome features are interdependent (covariates) or affected by sampling bias, we used a multivariate analysis framework to disentangle habitat properties from phylogenetic inertia. This allows for the quantification of environmental and phylogenetic factors that impact diversity within species. To construct the phylogenetic tree, we used the concatenated protein sequences of 40 conserved universal marker genes which were aligned using the ClustalOmega aligner (default parameters). The tree was constructed using FastTree2 (JTT model) [52].

Pangenome features are generally expected to be phylogenetically conserved as a result of the evolutionary history of a given species (phylogenetic inertia), and predefined by past exposures to different environments. A prominent example of phylogenetic inertia is the observation that closely related species tend to share more genes, i.e., gene content similarity follows phylogeny [2, 17]. Further, habitat preferences are also phylogenetically predetermined [18] and dispersal capability varies across different taxa [19, 20]. On the other hand, environmental factors shape genome architecture and the pangenome in general [21]. A (pan)genome’s functional potential mirrors both niche and phylogenetic signals [22] and consequently, phylogenetic relatedness and genome functionality are thought to be mildly predictive of species ubiquity and genome size [23–25]. Thus, it is expected that variation among pangenome features is associated with both phylogenetic inertia and environmental preferences. Yet, as phylogeny and habitat preferences are themselves correlated, their interactions need to be considered (Fig. 1).

The pangenome concept and its derivative measures (features) have been used extensively in the field of comparative genomics of prokaryotes to: (i) define species boundaries [26, 27], (ii) describe the genomic diversity of species [28], (iii) reveal origins of mutualistic and pathogenic strains [16] and (iv) characterize evolutionary and ecological mechanisms that shape genome architecture [8, 29, 30]. Here, to explore the general principles of pangenome evolution and to disentangle the differential impact of environment and phylogeny, we performed an analysis of over 7,000 high-quality genomes, encompassing 155 prokaryotic species from 10 phyla and 83 environments (Fig. 1). We computed 21 established pangenome features. The variation across these features was explored with respect to phylogenetic inertia and environmental constraints/preferences (characterized by 83 habitat descriptors) of the studied species. Using this framework, we quantified interdependencies of pangenome features, identified novel relationships among them, and estimated how habitat and phylogeny shape pangenome architecture. Within our dataset we could attribute up to 67% of the variation of pangenome features to habitat and phylogeny, which holds when scaling up to 10,100 species.

## Methods

### Genomic data

In this study we used 7,104 genomes from 155 consistently defined species (defined using 40 universal marker genes—specI clusters [31]) obtained from the proGenomes database [32] (see Supplementary Table 2). This removes biases resulting from differing species definitions in distinct research areas. To further increase the reliability of further analysis, we included only high-quality genomes with 300 or fewer contigs. Only one genome from any pair of genomes was retained for downstream analysis when pairwise nucleotide identity in the core-genome was 100% and pairwise gene content overlap (Jaccard index) >99%. We used only species that contained at least ten high-quality genomes in the proGenomes database [32]. Further, we compiled two confirmatory datasets that included species for which less than ten genomes were sequenced. The first confirmatory set represents the full proGenomes database (the same database underlying the pangenome dataset) consisting of 4,582 species (24,223 high-quality genomes). The second dataset represents the full proGenomes2 database (a recent update of proGenomes) of 84,022 high-quality genomes from 10,100 species [33]. For our confirmatory analyses, we computed the average genome size for each species within each of the datasets.

### Habitat annotation

Habitat metadata for isolates/strains were obtained from the PATRIC database [34], the Microbe Atlas Project database (https://microbeatlas.org) and Global Microbial Gene Catalog (http://gmgc.embl.de*)*, resulting in the reliable annotation of species to one or more habitats (83 total habitats, see Supplementary Table 3). PATRIC annotations were manually curated by searching for a predefined list of keywords (Supplementary Table 1). Any given species was considered present in the habitat from Global Microbial Gene Catalog if at least ten genes of a pangenome where present in a sample from that habitat. To annotate environmental preferences using the Microbe Atlas Project dataset we extracted 16S rRNA genes (at least 50% of the entire gene length) from the original genbank files or, if these annotations were missing in the genbank file, we re-annotated the genomes rRNA genes using barrnap [35]. Extracted 16S rRNA sequences were then mapped to the Microbe Atlas Project reference database using MAPseq [36] to link species clusters to Operational Taxonomic Units at 98% sequence similarity. Associations between each species and their potential habitats from the Microbe Atlas Project where tested for significance using Fisher’s Exact Tests (Benjamini-Hochberg correction, *p* ≤ 0.05). Ubiquity was estimated as the sum of all positive associations across all habitats in the Microbe Atlas Project dataset. The final annotation is available as Supplementary Table 3.

### Pangenome reconstruction

**Fig. 2:**
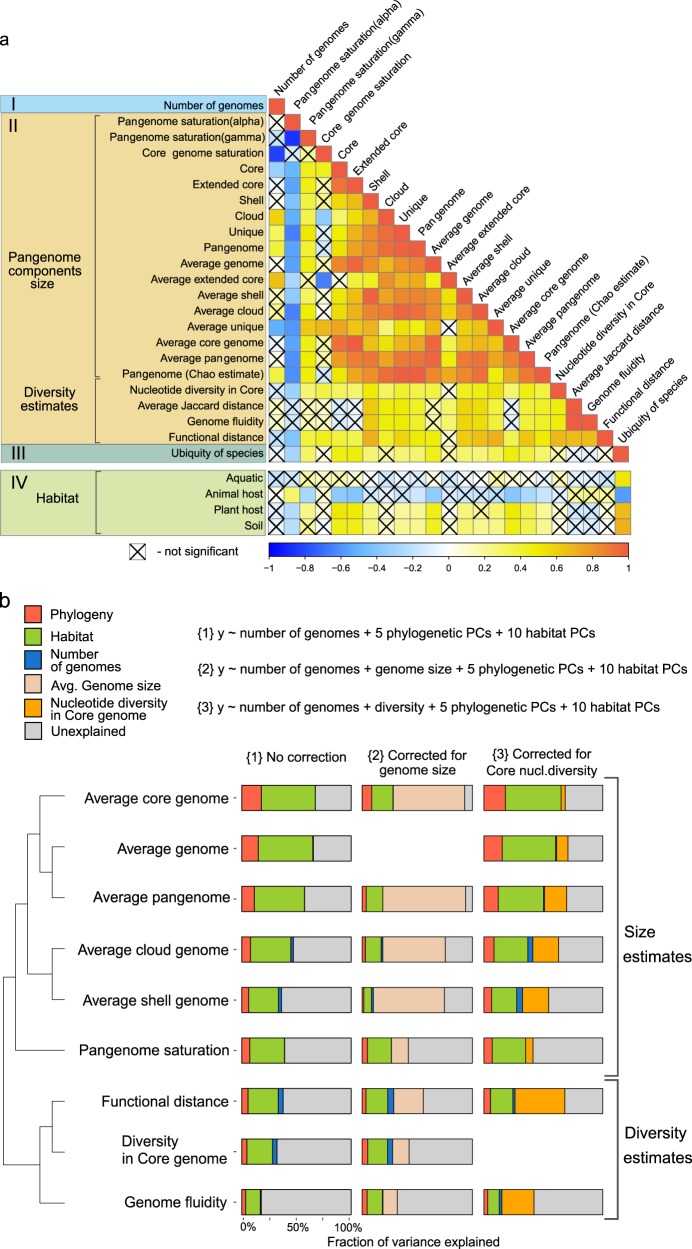
Relationship between different pangenome features. **a** Correlation matrix between (I) the number of conspecific genomes used to estimate pangenome features, (II) 21 pangenome features, (III) the ubiquity of species as an environmental feature computed from habitat preference of strains, and (IV) major habitat groups from the Microbial Atlas project. The heatmap visualizes Spearman Rho values for correlations between sample size (I), 21 pangenome features (II), and species ubiquity (III). Four major habitats (aquatic, animal host, plant host, soil (IV)) were correlated to the (I) number of conspecific genomes, (II) pangenome features, and (III) ubiquity via point-biserial correlation. Statistical significance of correlations was determined using adjusted *p* values (using Benjamin-Hochberg correction) <0.05. **b** Clustering of a subset of nine pangenome features based on their pairwise correlation strengths. Horizontal stacked charts present amount of variance explained by various predictors (number of genomes, phylogeny, and habitat represented by their principal components (PCs), and genome size or diversity). The first set of stacked charts (“no correction”) shows variance explained in pangenome features by the number of genomes used to compute pangenome features as well as species’ phylogeny and habitat preferences; the second and the third sets of stacked charts represent the amount of variance explained (see “Methods”) by the same set of predictors when correcting for genome size or nucleotide diversity in core-genome respectively. Size and diversity estimates form distinct feature groups.

Pangenomes for the 155 species studied were constructed using the Roary pipeline [37]. Input genomes for pangenome construction were first annotated using Prokka [38]. We identified homologous gene clusters at an amino acid identity threshold of 80% [39–42]. Pan and core-genome curves were generated via 30 input order permutations (similar to the approach in the GET_HOMOLOGUES pangenome pipeline [43]). Fitting of non-linear regressions was performed in R v.3.3.2 [44] using the “nls package” [45]. The total number of genes in the pangenome of a given species, the number of new genes added per genome and the total number of core genes were modeled using Eqs. (1, 2, and 3) respectively to estimate the openness of pangenomes [6, 7].

1 $$G \,=\, kN^\gamma \,+\, c,$$

2 $$G \,=\, kN^{ - \alpha },$$

3 $$G \,=\, ke^{ - N \ast \gamma } \,+\, c,$$

where *G*—number of genes; *N*—genome number that is added to analysis; k, c,—constants; *α* and *γ*—saturation coefficients. When *γ* ≤ 0 in Eq. (1)—pangenome is closed (saturated) (Fig. 2a); 0 < γ ≤ 1—pangenome is open (Fig. 2a). When *α* < 1 in Eq. 2—pangenome is open, *α* > 1—pangenome is closed.

Classification thresholds for pangenome subcomponents were defined as follows: core genes–present in all strains; extended core—present in >90% of genomes; cloud genes—present in <15% (includes unique genes in pangenome); the remaining part of pangenome was considered “shell” genes (Supplementary Fig. 1). These thresholds are based on default parameters of the Roary pipeline [37], although we readjusted the extended core threshold to 90%, as suggested by the distribution frequency of genes within the pangenomes in our dataset (Supplementary Fig. 2). The R package “micropan” [46] was used to compute genomic fluidity [47], Chao’s lower bound for gene content in the pangenome [48] and Heaps’ alpha Eq. (2) [6]. Functional distance between strains within each pangenome was estimated as Jaccard distance based on eggNog v4.5 annotations [49] of pangenome gene clusters. Twenty-three parameters (21 pangenome features, plus the number of conspecific isolates and species ubiquity) were compared using Spearman’s rank correlation to investigate the relationship between sample sizes, subcomponents of pangenome, saturation parameters (*γ* and *α*) from Eqs. (1, 2, and 3), genome fluidity functional distance and core-genome nucleotide identity (see Supplementary Table 1 for definitions of pangenome features). To obtain unbiased estimates of core and pangenome sizes we calculated average core and pangenome sizes across 30 random combination of nine genomes for each species (also see Supplementary Table 1 and Supplementary Fig. 1). Hierarchical clustering of a subset of pangenome features was performed on absolute values of pairwise Spearman Rho values as displayed in Fig. 2a.

### Phylogenetic signal and phylogenetic generalized least squares

An approximate maximum likelihood phylogenetic tree of all 155 species was generated using the *ete-build* concatenation workflow “clustalo_default-trimal01-none-none” and “sptree-fasttree-all” from ETE Toolkit v3.1.1 [50], using protein sequences of 40 conserved universal marker genes [31, 51, 52] and default parameters for the ClustalOmega aligner [53] and FastTree2 [54] with the JTT model [55].

To estimate the phylogenetic signal of genomic traits, we used the R package “phylosignal” [56] with Pagel’s Lambda [57], following guidelines for phylogenetic signal analysis [58, 59] (Supplementary Fig. 3). The “Caper” R package was used for phylogenetic generalized least squares regression [60].

### Quantification of explained variance in pangenome features

The cophenetic distance matrix obtained from the phylogenetic tree and the binary habitat association matrix (83 habitats in total) were each decomposed using the “FactoMineR” R package [61]. The first five phylogenetic principal components (PCs) (accounting for ~80% of phylogenetic variance) and ten habitat PCs (accounting for ~50% of habitat variance) were used for variance partitioning. PCs were selected using the “broken stick” model [62]. The first two PCs for phylogenetic and habitat matrices decompositions are visualized in Supplementary Figs. 4 and  5. In order to minimize the impact of differential sampling size, the number of genomes used for each species was included as an additional variable. The fraction of the variance explained by habitat and phylogeny were estimated using the CAR metric which performs a decorrelation of predictors [63] implemented in the “car” R package with the following models:

4 $${\mathrm{Pangenome}}\;{\mathrm{feature}} \,=\, {\mathrm{number}}\;{\mathrm{of}}\;{\mathrm{genomes}}\;{\mathrm{in}}\;{\mathrm{each}}\;{\mathrm{species}} \\ +\, 5\;{\mathrm{phylogenetic}}\;{\mathrm{PCs}} \,+\, 10\;{\mathrm{habitat}}\;{\mathrm{PCs}}$$

5 $${\mathrm{Pangenome}}\;{\mathrm{feature}} \,=\, {\mathrm{number}}\;{\mathrm{of}}\;{\mathrm{genomes}}\;{\mathrm{in}}\;{\mathrm{each}}\;{\mathrm{species}} \\ +\, {\mathrm{genome}}\; {\mathrm{size}} \,+\, 5\;{\mathrm{phylogenetic}}\;{\mathrm{PCs}} \,+\, 10\;{\mathrm{habitat}}\;{\mathrm{PCs}}$$

6 $${\mathrm{Pangenome}}\;{\mathrm{feature}} \,=\, {\mathrm{number}}\;{\mathrm{of}}\;{\mathrm{genomes}}\;{\mathrm{in}}\;{\mathrm{each}}\;{\mathrm{species}} \\ +\, {\mathrm{core}}\;{\mathrm{genome}}\;{\mathrm{nucleotide}}\;{\mathrm{diversity}} \\ +\, 5\;{\mathrm{phylogenetic}}\;{\mathrm{PCs}} + 10 \;{\mathrm{habitat}}\;{\mathrm{PCs}}.$$

We also performed the model-fitting procedure (Eq. 4) on 1,000 permutations of the first five phylogenetic PCs and first ten habitat PCs to ensure that the actual habitats and phylogeny data explained a higher fraction of the variance than randomized models (Supplementary Fig. 6).

## Results

### Delineation of pangenomes and habitats descriptors

The basis of this study is a large collection of pangenomes from a diverse set of prokaryotic species. To establish this collection, we filtered the proGenomes database of annotated prokaryotic genomes [32] to select consistently defined species (see “Methods”, also [31]) for which at least ten high-quality genomes (conspecific isolates/strains/genomes; further referenced as strains or genomes) were available (Fig. 1, also see “Methods”). For each of the resulting 155 species, we computed 21 pangenome features (ranging from pangenome saturation to functional distance, see Fig. 2a and Supplementary Table 1). These features have been shown to characterize different aspects of the pangenome structure and have been previously used in pangenome analyses of individual microbial species [6, 47]. Partitioning the pangenome into subcomponents (“core”, “shell”, “cloud”; see “Methods”) enabled us to relate the evolutionary adaptations of core and accessory genome features to environmental pressures separately. Pangenome subcomponents varied in size, for example, average core-genome size was in the range of 443–5,964 genes; average pangenome size—959–17,739 genes; average shell 18–2,409; average cloud—5–839 genes. We further annotated all genomes and species with regards to their habitat preferences. Yet, environmental metadata for many isolates and prokaryotic species are incomplete and biased towards clinically relevant host-associated annotations, leaving the ecological niches of many species under-characterized. To improve habitat assignments, we used multiple, conceptually different habitat databases. More specifically, we merged the information obtained from the PATRIC database [34], the Microbial Atlas Project database (http://devel.microbeatlas.org/) and the Global Microbial Gene Catalog (http://gmgc.embl.de). This resulted in detailed and accurate habitat annotations using 83 habitat descriptors (see “Methods” and Supplementary Table 3). On average, each species was present in 16.5 ± 7.8 (out of 62 possible) habitats in Microbial Atlas Project; 2.4 ± 1.1 (out of 5 possible) from manually curated PATRIC habitat annotations; and 3.6 ± 2.8 (out of 16 possible) in the Global Microbial Gene Catalog (Supplementary Table 3).

### Interdependencies of pangenome features

The relationships between different pangenomes features can be an indication of similar evolutionary pressures acting on the related features. Further, correlations between different features can decrease the accuracy of analyses when not considered. The number of genomes used to infer a species’ pangenome needs to be accounted for as it can potentially influence the calculation of some of these features. Hence, we estimated interdependencies for (i) the number of conspecific strains (the number of genomes per pangenome), (ii) the 21 computed pangenome features, (iii) species ubiquity, and (iv) habitat preference (see Supplementary Table 3 for estimates of pangenome features, Supplementary Table 1 for definition and Supplementary Table 4 for correlation summary) (Fig. 2a). Estimates of pangenome size and the size of its components (core, shell, and cloud) are strongly correlated with each other (Fig. 2a). As expected, mean genome size strongly correlated with several features, including core-genome size (Spearman Rho = 0.955, *p* < 0.00001), pangenome size (0.963, *p* < 0.00001), and core-genome nucleotide diversity (0.373, *p* = 0.00003), indicating that a species’ average genome size is highly predictive of its pangenome features, especially pangenome size. While these results confirm the accuracy of our methodology, we found some pangenome features to be unreliable due to their observed associations with sample size (number of conspecific strains). Significant correlations were found for core-genome saturation, core-genome size, total pangenome size, as well as the sizes of “cloud” and “unique genes”, indicating that sampling biases might affect these features. Hence, we excluded these features from our in-depth analyses. For pangenome and core-genome sizes, we used average normalized size features instead (average of 30 random combinations of nine genomes per species, see Supplementary Table 1 and Supplementary Fig. 1).

Among the reliable features, we unexpectedly found the several pairs of conceptually related pangenome features, which were not correlated. For example, the relationship between genome fluidity [47] and pangenome saturation was not significant (Spearman Rho = 0.15, *p* = 0.72), despite the fact that both measures are commonly used to estimate the openness of pangenomes [8, 47] (Supplementary Table 1). This might indicate that these two measures characterize different aspects of pangenome openness. Previous studies have hypothesized an implicit sampling bias as a possible explanation for this observation [47], but we did not detect a significant relationship with the number of sampled genomes in our large dataset for either of the two features.

Furthermore, the average pairwise functional distance (average Jaccard distance based on orthologous groups) between conspecific strains positively correlated to the vast majority of pangenome features (Fig. 2a). Only three pangenome features were not significantly correlated to the average pairwise functional distance, namely the size of the extended core, the number of conspecific strains (number of conspecific genomes used to compute pangenome features) and ubiquity (see Supplementary Table 4 for Spearman Rho and *p* values). We further found that species with larger genomes tend to have a higher functional diversity (Spearman Rho = 0.48, 6.5e–9), mainly driven by changes in the size of the pangenome shell. This seems to imply that functional diversity is maintained within a substantial fraction of organisms in species with larger genomes.

To study which factors shape pangenome features, we performed variance partitioning on 9 out of 21 features representing qualitatively different pangenome properties that are practically unaffected by sample size (nonsignificant correlations with Spearman Rho close to 0, see Fig. 2a). We explored the interdependencies of these nine pangenome features by clustering them according to their correlation strengths and identified two subgroups (Fig. 2b, see also Supplementary Table 5). These subgroups split the features into diversity estimates (core-genome nucleotide diversity, functional distance, and genome fluidity) and size estimates (average genome, pangenome, core, shell, and cloud) implying differing evolutionary dynamics for these feature groups. Specifically, size-related pangenome features were better explained by phylogenetic and environmental preference compared with diversity estimates (Fig. 2b). We also show that, after correcting for within-species diversity, a substantial amount of variance is still explained by environmental preferences and phylogeny (Fig. 2b). These observations are highly relevant for understanding the adaptiveness and evolution of pangenomes, which have been under ongoing discussion [8].

### Species ubiquity is related to core-genome size

**Fig. 3:**
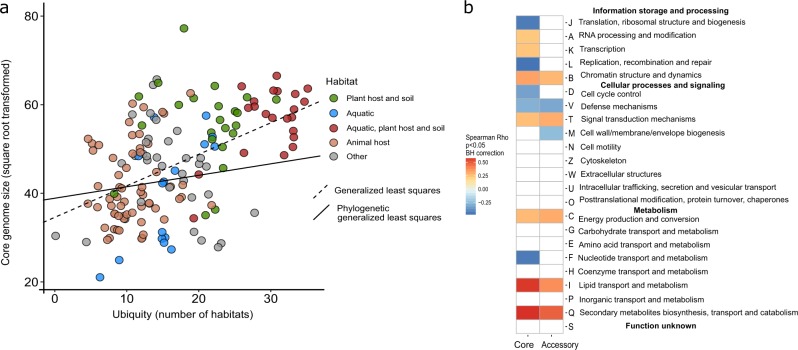
Effect of ubiquity on core-genome size and functional content. **a** Species ubiquity (number of habitats a species was assigned to), a habitat feature, is linked to core-genome sizes after correction for phylogenetic effect (Phylogenetic generalized least squares, *p* value = 0.00005, *λ* = 0.98 (95% CI 0.957, 0.992), partial *R*-square (for ubiquity coefficient) 0.09, see also Supplementary Table 6). **b** Correlation of ubiquity with the relative frequency of functional categories (COG categories assigned by eggNog v4.5 [47]) in core and accessory genomes. Species of high ubiquity tend to encode more proteins involved in lipid metabolism (I) and secondary metabolite biosynthesis (Q).

All surveyed species are present in multiple habitats (Supplementary Table 3) and the transition between free-living and host-associated lifestyles were observed frequently on both micro- and macro-evolutionary (and ecological and evolutionary) timescales, imposing multidirectional pressures on the evolution of their genome architecture [64]. Species ubiquity is a potentially important factor contributing to the evolution of specific pangenome features that needs to be considered, because species with broad ecological niche are likely to have different evolutionary constraints compared with specialists [65]. We operationally defined species ubiquity as the sum of all positive associations with each habitat in the Microbe Atlas Project dataset (see “Methods”), which provides the most comprehensive habitat annotations for our datasets. Broader ecological niches and higher ubiquity have been suggested to be associated with larger and more functionally versatile genomes [66]. Therefore, we investigated the relationship between the ubiquity of each species with its pangenome features in depth and found several associations (Fig. 2a). We observed a moderate, but significant association of species ubiquity (Fig. 3a) with average normalized core-genome size (average core-genome size of random combinations of nine genomes, Supplementary Fig. 1) and pangenome saturation. Other pangenome features were not correlated with ubiquity after correcting for phylogenetic effects (Fig. 3a, Supplementary Table 6). This suggests that a larger core-genome may be important to facilitate persistence and proliferation in multiple habitats. The core-genome of highly ubiquitous species was enriched in genes coding for proteins involved in lipid metabolism and secondary metabolite biosynthesis (COG categories I and Q in Fig. 3b, respectively). This is congruent with earlier studies, suggesting that secondary metabolite biosynthesis might be implicated in adaptation to multiple environments [66].

### Dissecting the impact of phylogenetic inertia and environment on pangenome features

Phylogenetic inertia and habitat are thought to have a substantial impact on genome evolution [67, 68], yet to which degree different aspects or features of pangenomes are affected is unknown. Our analysis framework allows us to study these associations in depth. Hence, we quantified differential contributions of phylogenetic and environmental factors to pangenome architecture. Pangenome features were modeled as combinations of the number of conspecific genomes considered, phylogenetic placement, and habitat preference. For this we used an abstract representation of phylogeny and habitats as PCs, accounting for dimensionality, collinearity, and redundancy within these data. The respective relationships were approximated using a linear model (see “Methods”), which allowed us to estimate the variance of pangenome features between species explained by phylogenetic effect and habitat preferences:

$${\mathrm{Pangenome}}\;{\mathrm{feature}} =\, {\mathrm{Number}}\;{\mathrm{of}}\;{\mathrm{genomes}} \\ +\, \left[ {{\mathrm{Genome}}\;{\mathrm{size}}\;{\mathrm{or}}\;{\mathrm{diversity}}} \right] \, +\, 5\;{\mathrm{phylogenetic}}\;{\mathrm{PCs}} \\ +\, 10\;{\mathrm{habitat}}\;{\mathrm{PCs}}.$$

**Fig. 4:**
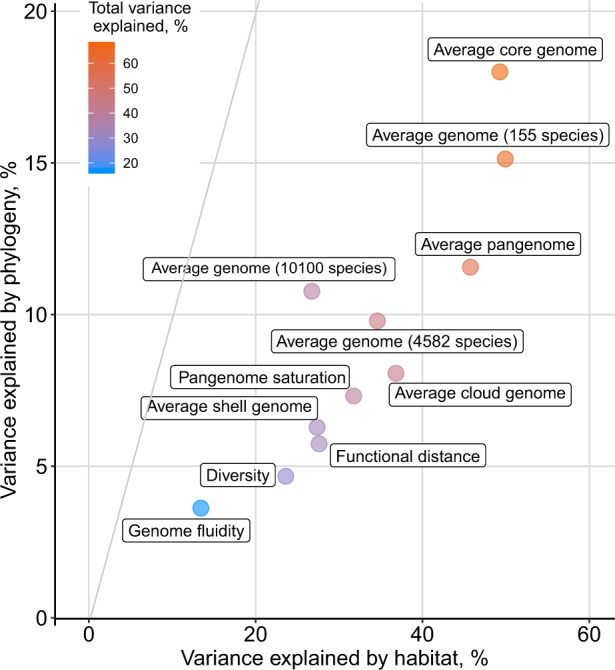
Partitioning of variance in pangenome features explained by phylogenetic inertia and habitat preferences (*R*-square (car score)) based on model {1} from Fig. 2b.

Together, habitat and phylogenetic effects explained the large parts of the variance (up to 49% by habitat and 18% by phylogenetic effect) in all selected features (Fig. 2b, Supplementary Table 5). This remained true, even when controlling for genome size or core-genome diversity (as evident when these were included in the model as predictors as in the second and third set of stacked charts of Fig. 2b) (Supplementary Table 6). Habitat and phylogeny have considerable independent effects on pangenome features, although the impact of habitat preferences was consistently stronger (Fig. 4). Diversity estimates, in contrast, were explained to a lesser degree by habitat preferences of species and phylogenetic inertia, as they likely reflect spatio-temporal (microevolutionary) variation of subpopulations within-species due to local adaptation and/or genetic drift [28, 69]. For example, a higher fraction of core-genome size (and genome size) variance was explained by species habitat preference than any other pangenome feature (including accessory genome size when considered separately), implying that core-genome size might be linked to a species’ ecology while the accessory genome might often be more affected by random gene acquisition via horizontal gene transfer and loss [70–73]. The observed signals were robust to technical and annotation noise, as random permutations of habitat and phylogenetic PCs did not exceed the observed data in variance explained (except for genome fluidity (Supplementary Fig. 6)). The strongest phylogenetic effects were observed for average core, pangenome, and genome sizes (confirmed using Pagel’s Lambda estimate to test the strength of the phylogenetic signal [57] (Supplementary Fig. 3). Overall, up to 67% of the variance of different pangenome features was explained by habitat and phylogeny (Figs. 2b and  4). Notably, habitat preferences and phylogenetic inertia affected diversity- and size-based pangenome features differentially (Fig. 2b).

Due to the required number of genomes of computing pangenomes, species from just ten phyla were part of the pangenome study. To confirm our results on the impact of phylogenetic inertia and habitat preferences on bacterial evolution across a larger phylogenetic range, we used the full proGenomes dataset (4,582 species, ca. 24,000 genomes from 59 phyla, see “Methods” and Supplementary Table 7) and the even larger proGenomes2 dataset (10,100 species, ca. 84,000 genomes from 59 phyla, see “Methods” and Supplementary Table 8). For most species in these datasets only one or a few genomes were available, which did not allow for the computation of pangenomes. Hence, we leveraged our observation that the average genome size of prokaryotic species is strongly correlated to various pangenome features (Fig. 2). In consistence with the pangenome dataset, habitat preference had a much greater effect (Fig. 4; proGenomes: 34.6% variance explained; proGenomes2: 26.7%) than phylogeny (Fig. 4; proGenomes: 9.8% variance explained; proGenomes2: 10.8%). The slightly lower amount of variance explained in the larger datasets might be due to habitat annotation ambiguity and phylogenetic uncertainty. Yet, it confirms that habitat has a larger impact than phylogeny on pangenome architecture (Fig. 3a).

### Environment-driven, convergent evolution of pangenome features

**Fig. 5:**
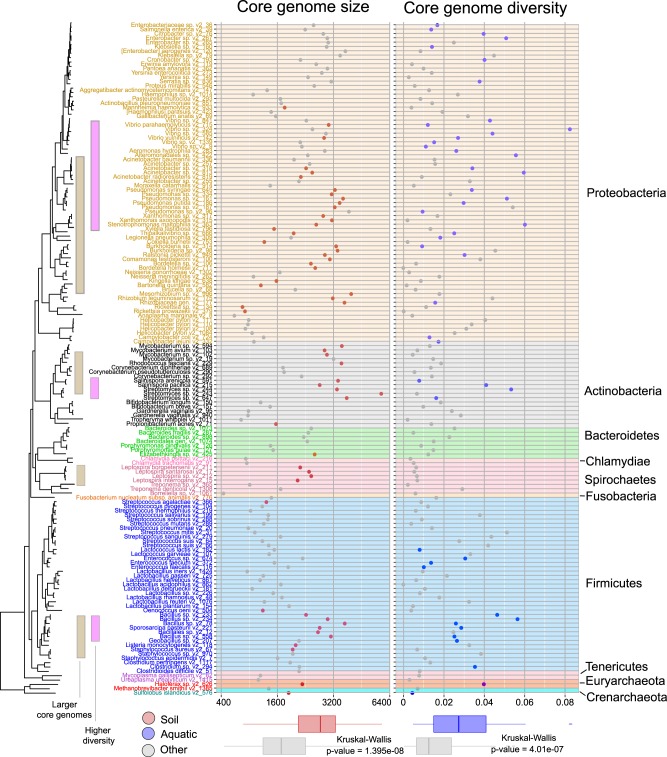
Phylogenetic tree of 155 microbial species with scatter plots of core-genome size and average nucleotide diversity of core genomes. Soil-associated species tend to have larger core genomes (marked in red in the left scatter plot), aquatic species tend to be more diverse (marked in blue in right scatter plot). Tree labels and background of scatter plots are colored by their taxonomic annotations (phylum). Bottom panel: Relationships between habitats and core-genome size and average nucleotide diversity of core genomes.

To investigate how habitat preferences and phylogenetic inertia impact bacterial evolution in more details, we next analyzed the effects of selected major habitat groups (soil-associated, aquatic, animal-host-associated, and plant-host-associated habitats) on the sizes of genomes/pangenomes and within-species diversity, accounting for their phylogenetic background (Fig. 2a). As expected, soil and plant-host habitats were associated with larger pan and core genomes, while animal host habitats were associated with smaller ones [16, 74]. Aquatic habitats were not a good predictor for size-related pangenome features, which might be indicative of their heterogeneous nature [21, 75]. The distribution of core-genome sizes across the phylogenetic tree of species studied showed that large core genomes have independently evolved (Kruskal–Wallis test, chi-squared = 32.194, df = 1, *p* value = 1.395e–08) in soil-inhabiting species from at least four (out of ten analyzed) phyla (Proteobacteria, Actinobacteria, Spirochaetes, and Firmicutes, Fig. 5). Small core-genome sizes independently evolved at least three times (Proteobacteria, Actinobacteria, and Firmicutes) in our dataset. Nucleotide diversity of the core-genome was, in contrast to size, less affected by habitat and phylogenetic signals (Fig. 4, Supplementary Fig. 3). Nevertheless, species with a higher nucleotide diversity within their core-genome were positively associated with aquatic habitats (Fig. 5) (Kruskal–Wallis test, chi-squared = 25.69, df = 1, *p* value = 4.01e–07), in line with earlier observations from metagenomics [18]. In conclusion, core-genome sizes and (to a lesser degree) diversity in prokaryotic species depend on broad habitat type(s) and range, implying that adaptation to a given habitat range might lead to convergent evolution towards habitat-specific core-genome sizes (e.g., soil-associated species have larger genomes, Fig. 5).

## Discussion

The question of how environments shape biological diversity is central to modern biology, extending beyond evolutionary biology. Microbial evolution is particularly affected by ecological constraints due to the broad distribution of microbial life across virtually all environments on Earth. Our understanding of microbial species and their evolution has been extended by the pangenome concept [5, 6]. By analyzing microbial pangenomes in the context of their environmental preferences and phylogeny, we were able to dissect major forces that shape microbial genomes. Our results suggest that habitat and phylogeny explain the majority of variation of pangenome features across different species, with differential contributions to size and population-level diversity measures. These results are highly important for an ecological understanding of prokaryotic evolution and this represents the first time that these factors are quantified in a natural setting. Nonetheless, different theories and concepts have been postulated to explain microbial evolution in response to the environment [8, 76]. For example, it has long been thought that a large pool of accessory genes would be beneficial in certain habitats (and habitat combinations). On the other side, the role of the pangenome as an adaptive evolutionary entity has been recently disputed. In the respective debate [8, 77–82], analyses of pangenome size estimates (Fig. 2b) have led to the conclusion that pangenomes are adaptive [8], while studies focusing on diversity measures such as genome fluidity led to the conclusion that pangenome evolution is predominantly neutral [77]. Our analysis shows that environmental conditions and phylogenetic inertia affect size-related pangenome features to a higher degree (than diversity features), suggesting that the adaptiveness of pangenomes is at least partially explained by environmental preferences of species and their phylogenetic inertia (Fig. 2b). Mechanistically, it is likely that ecological constraints imposed by habitats drive pangenome evolution, through natural selection, genetic drift, and/or both and most likely in dependence on the species’ effective population size [83]. Yet, pangenome size and other features are also partially determined by phylogenetic inertia: we observed that core-genome size and average genome size (number of protein-coding genes) were most affected by phylogenetic position (Figs. 2b, 4). The conservation of the core-genome in a given clade is likely due to the fact that it consists of essential genes that are under strong negative selection pressure [73, 84, 85], which leads to vertical “heritability” of its content and size from ancestral species to descendants during speciation events.

Building on a previous study, which showed a weak positive relationship between the ubiquity of species and overall genome size [66], we found that the strongest (albeit still moderate) correlation was with core-genome size though a larger accessory genome had been thought to be instrumental for species ubiquity [66, 76]. Our more detailed observations suggest that genes that facilitate ubiquity (i.e., species presence across many habitats) are usually present in the core-genome, which is further supported by the absence of a significant correlation between average intra-species pairwise functional distance and ubiquity (Fig. 2a). If functional diversity of accessory genome was highly important for ubiquity, we would expect a positive correlation between intra-species pairwise functional distance and ubiquity. In other words, the expansion of a species into additional habitats requires almost all strains to have genes that facilitate survival and proliferation in all or most species habitats.

Overall, our results indicate important relationships between the environment, macro- and micro-evolutionary patterns in pangenome features, exemplified by the association between ubiquity and core-genome size. Hence, multifeature predictive modeling is able to predict the ubiquity and environmental preferences of microbial species from pangenome information and phylogenetic placement, whereby accuracy will increase as more (pan)genomes become available. Functional knowledge of the genes within the pangenome will also help to predict habitat ranges as well as required or desired environmental conditions, in the context of the respective phylogenetic placements.

## Supplementary information

Supplementary table 1

Supplementary table 2

Supplementary table 3

Supplementary table 4

Supplementary table 5

Supplementary table 6

Supplementary table 7

Supplementary table 8

Supplementary Figures

Tree 155 species

Tree 4582 species

Tree 10100 species
