# Supplementary material for: Disentangling the impact of environmental and phylogenetic constraints on prokaryotic within-species diversity
Source: ISME J. 2020 Feb 11;14(5):1247–59. doi: 10.1038/s41396-020-0600-z (PMC7174425; doi:10.1038/s41396-020-0600-z)
Supplement: Supplementary file 1 — Supplementary table 1 [file 41396_2020_600_MOESM1_ESM.docx]

| Supplementary Table 1. Definitions of pangenome features and other terms used in the manuscript; key-words used for habitat annotation from PATRIC database. | |
| --- | --- |
| Term | Definition |
| Number of genomes (sample size) | Number of genomes (also referred as sample size) that were used to calculate pangenome features for each species |
| Pangenome saturation (alpha) | Absolute value of alpha in equation [1] in methods |
| Pangenome saturation (gamma) | Coefficient gamma from equation [2] in methods |
| Core genome saturation | Coefficient gamma from equation [3] in methods |
| Pangenome | Total number of protein coding gene clusters estimated present in any number of genomes for each species (all genomes of a given species used to calculate this) |
| Core | Number of protein coding gene clusters found in every genome in a given species (all genomes of a given species used to calculate this) |
| Extended core | Total number of protein coding gene clusters in the extended core genome of a given species (all genomes of a given species used to calculate this) |
| Shell | Total number of protein coding gene clusters in the shell genome fo a given species (all genomes of a given species used to calculate this) |
| Cloud | Total number of protein coding gene clusters in the cloud genome for a given species (all genomes of a given species used to calculate this) |
| Unique | Total number of unique protein coding gene clusters found in a given species (all genomes of a given species used to calculate this) |
| Average genome | Mean number of protein coding gene clusters averaged across all genomes of each species |
| Average extended core | Mean number of protein coding gene clusters in the extended core averaged across all genomes of each species |
| Average shell | Mean number of protein coding gene clusters in the shell genome averaged across all genomes of each species |
| Average cloud | Mean number of protein coding gene clusters in the cloud genome averaged across all genomes of each species |
| Average unique | Mean number of unique protein coding gene clusters averaged across all genomes of each species |
| Average core genome | Mean number of protein coding gene clusters in the core genome averaged across 30 random combination of 9 genomes (for each species) |
| Average pangenome | Mean number of protein coding gene clusters in the core genome averaged across 30 random combination of 9 genomes (for each species) |
| Pangenome size (Chao lower bound estimate) | Chao lower bound estimate of the possible number of genes in a given species’ pangenome (calculated using the corresponding function of the "micropan" R-package) |
| Diversity | Mean nucleotide divergence (1 - nucleotide identity) of the core genome calculated across all pairs of genomes within a given species |
| Average Jaccard distance | Average Jaccard distance (1 - Jaccard index) calculated across all pairs of genomes within a given species |
| Genome fluidity | Genomic fluidity is calculated as the ratio of unique gene families over the sum of gene families averaged over randomly chosen pairs of genomes from within a given species of *N* genomes |
| Functional distance | COG-based average Jaccard distance between isolates/strains within a given species |
| Ubiquity of species | Ubiquity is the sum of all positive associations (Benjamini-Hochberg-corrected Fisher’s Exact Tests, p≤0.05) of a species with habitats in the Microbial Atlas Project dataset. In other words, ubiquity shows with how many habitats a certain species was associated. |
| Key-words for habitat annotation "soil" | ["terrestrial biome", "Terrestrial", "Soil", "soil", "Rhizosphere", "rhizosphere", "plant root", "Plant root", "root nodule", "Root nodule", "root nodules", "rhizosphere soils", "Tailings", "Rhizospheric", "rhizospheric", "Sand", "soil sample", "sediment", "Sediment", "Sludge", "sludge", "mud", "Mud", "Sand"] |
| Key-words for habitat annotation "aquatic" | ["Aquatic", "aquatic", "marine", "Marine", "water", "Water", "Sea water", "Fresh water", "sea water", "fresh water", "Pond", "pond", "river", "River", "lake", "Lake", "Ocean", "ocean", "creek", "Creek", "waterfall", "Waterfall", "Hot spring", "Hot springs", "hot springs", "Hot springs", "hot spring", "oceanic", "Oceanic", "sea ", "sea-", "Wastewater", "wastewater", "Rice paddies"] |
| Key-words for habitat annotation "host-associated" | ["HostAssociated", "Host-associated", "Host Associated", "host", "Host", "Rhizosphere", "rhizosphere", "Plants", "plant root", "Plant root", "root nodule", "Root nodule", "root nodules", "rhizosphere soils", "Rhizospheric", "rhizospheric", "Symbiotic", " skin", "Zoonotic", "rumen", "livestock-assoicated habitat", "Feces", "nasopharynx", "Blood", "sputum", "blood", "patient", "CSF", "stool", "feces", "Bodily fluid", "nares", "BLOOD", "Respiratory system"] |
| Key-words for habitat annotation "food" | ["food", "Food", "FOOD", "milk", "Milk", "cheese", "Cheese", "fermented", "meat", "Meat", "frozen peas", "dairy product", "dairy products", "Dairy", "koumiss", "burger", "yogurt", "Burger", "Yogurt", "wine", "Wine", "Champagne", "Cider", "champagne", "cider", "bread ", "Bread ", "liver paste", "liquor", "sashimi", "beef", "Beef", "Seafood", "seafood", "silage", "ground turkey", "chicken breast", "sourdough", "fermented soybean", "fermented", "fermentation", "Fermented", "Fermentation"] |
