# Supplementary material for: Disentangling the impact of environmental and phylogenetic constraints on prokaryotic within-species diversity
Source: ISME J. 2020 Feb 11;14(5):1247–59. doi: 10.1038/s41396-020-0600-z (PMC7174425; doi:10.1038/s41396-020-0600-z)
Supplement: Supplementary file 9 — Supplementary Figures [file 41396_2020_600_MOESM9_ESM.docx]

**Supplementary figures**

**
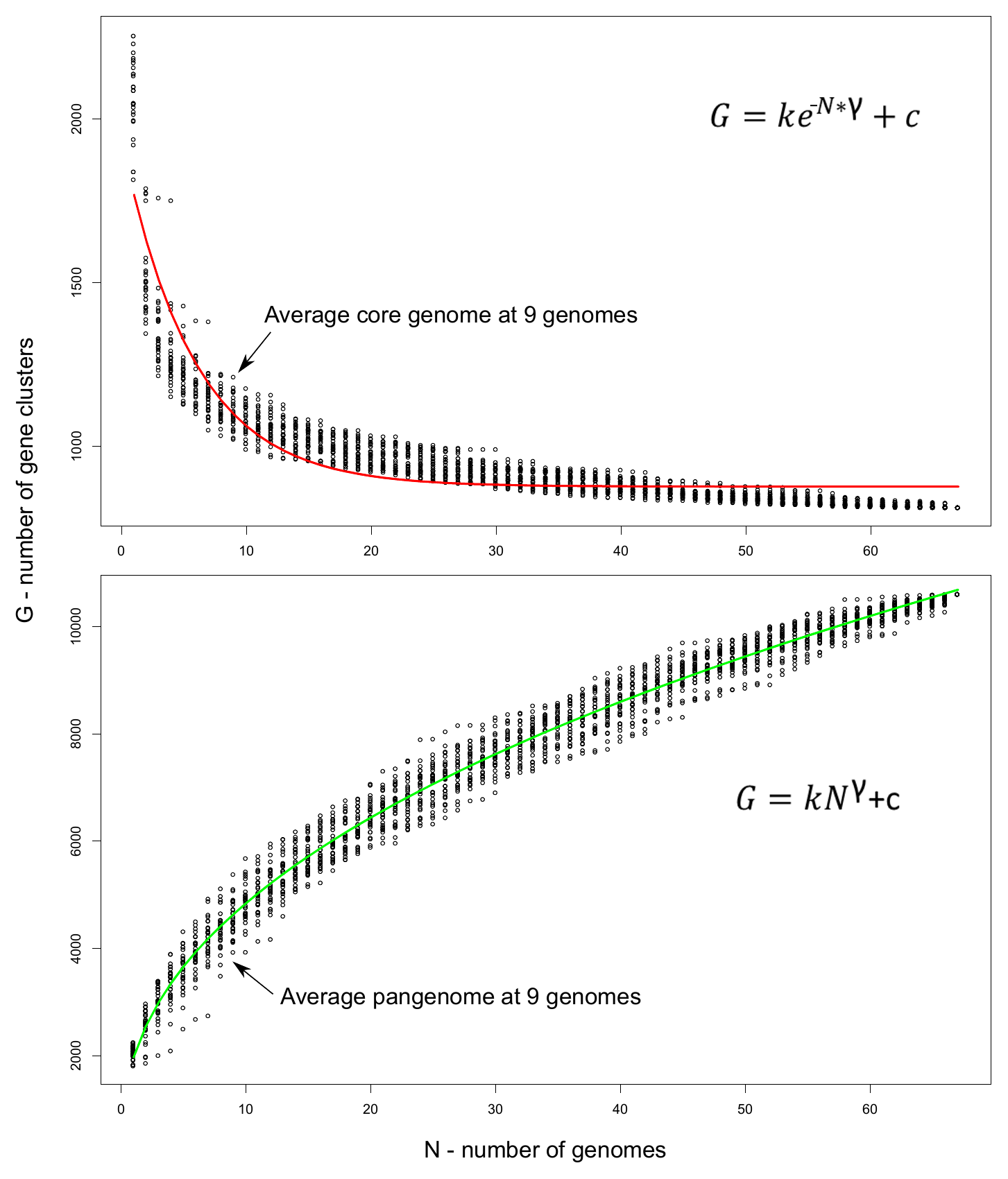
**

**Supplementary Figure 1.** Examples of saturation curves. Core- and pangenome size at a given number of (randomly chosen) genomes from a species. Fits to displayed formulas shown in red (core genome) and green (pangenome).


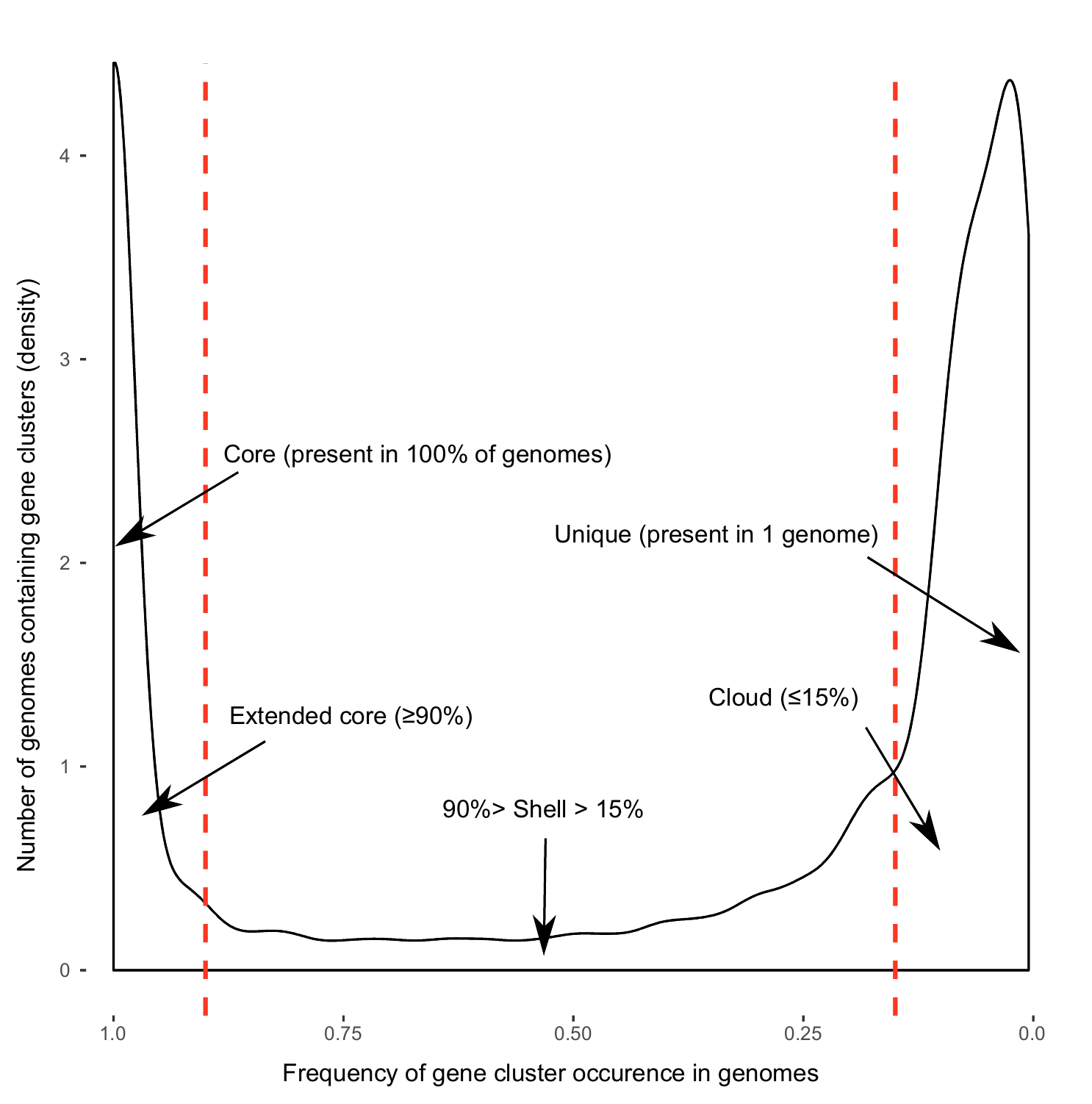


**Supplementary Figure 2.** Thresholds for pangenome components. Gene frequency distribution displayed in black. Dash lines represent boundaries between extended core and shell genome; shell and cloud genome.


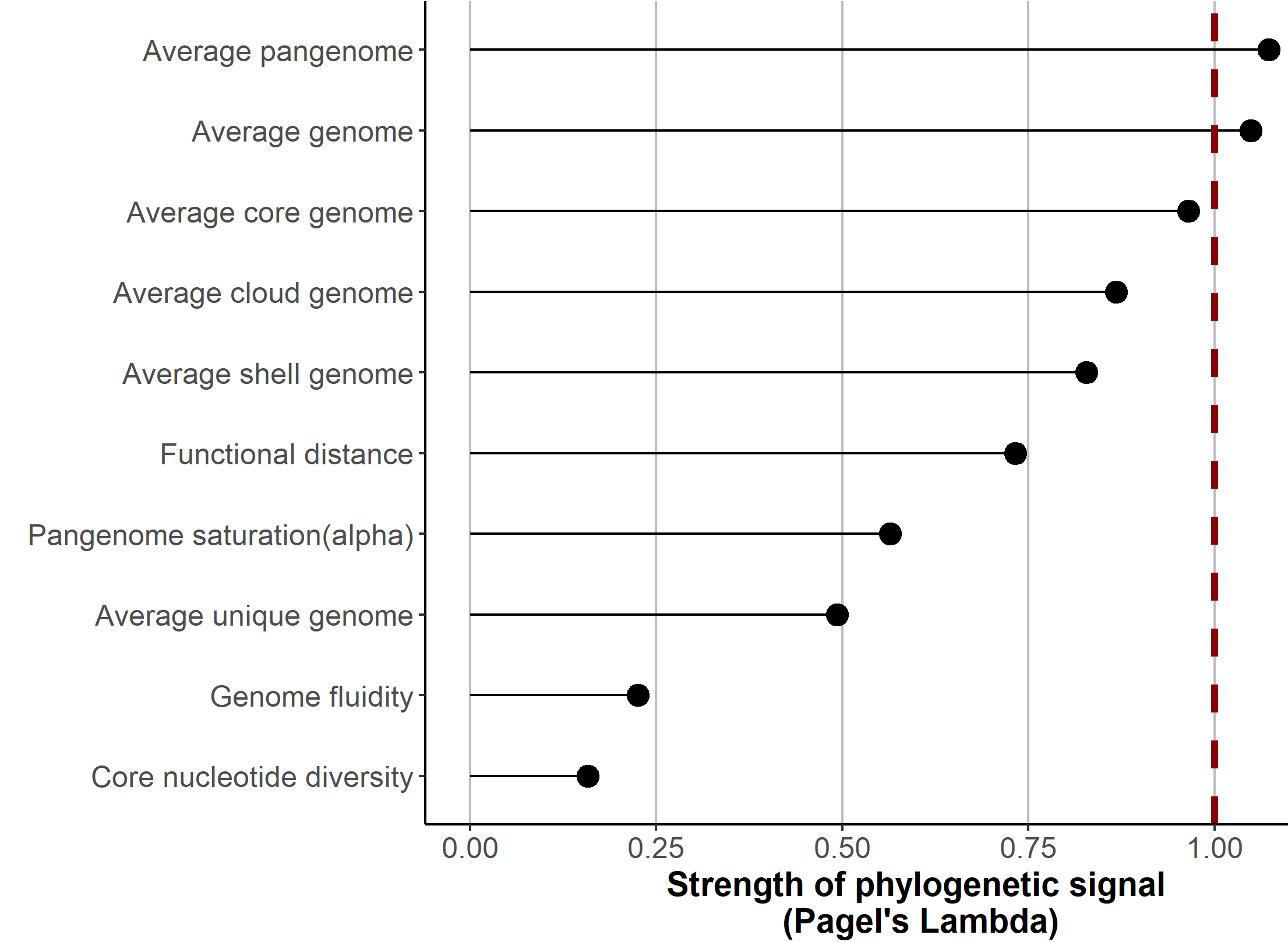


**Supplementary Figure 3**. Phylogenetic signal of 10 genomic characteristics across 155 species of Prokaryotes. When Pagel’s λ approximates to 1 – trait manifests phylogenetic signal (marked with dash line).


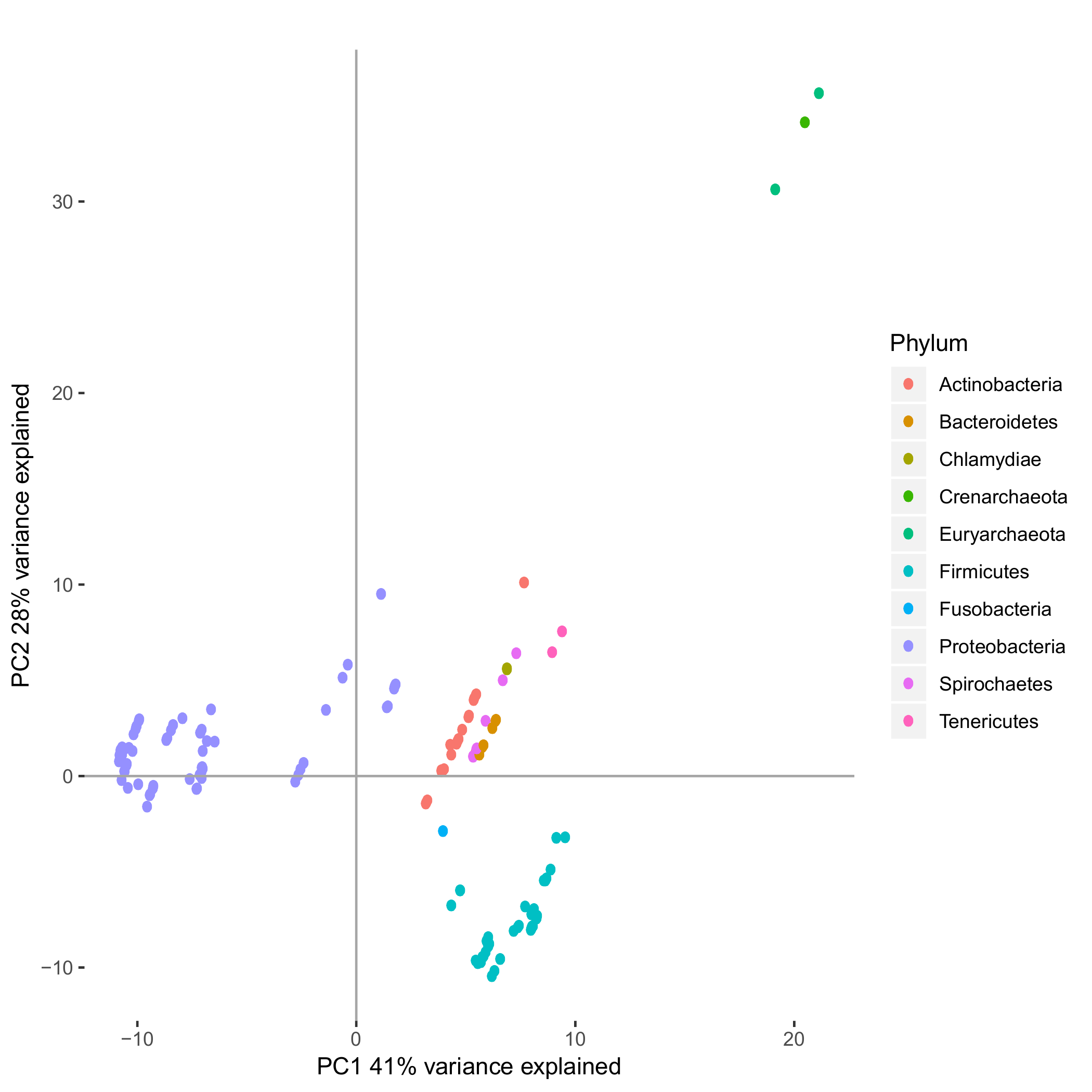


**Supplementary Figure 4.** Biplot of PCA (PC1 and PC2) using cophenetic distances observed in the phylogenetic tree reconstructed from 155 species used in this study.


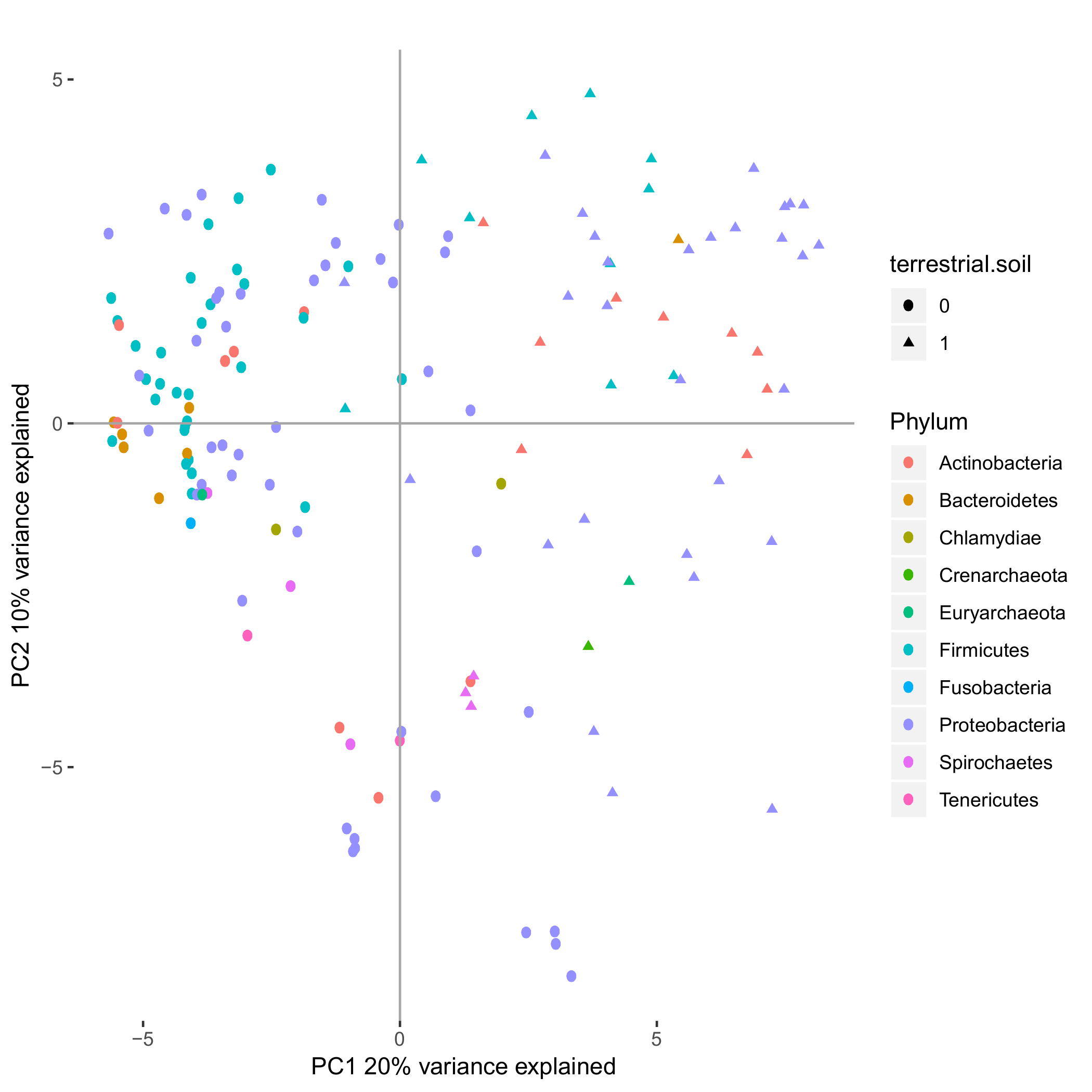


**Supplementary Figure 5.** Biplot of the first 2 principal components of the decomposition of the habitat-association matrix (0 - not associated with soil habitat, 1 - associated with soil habitat).


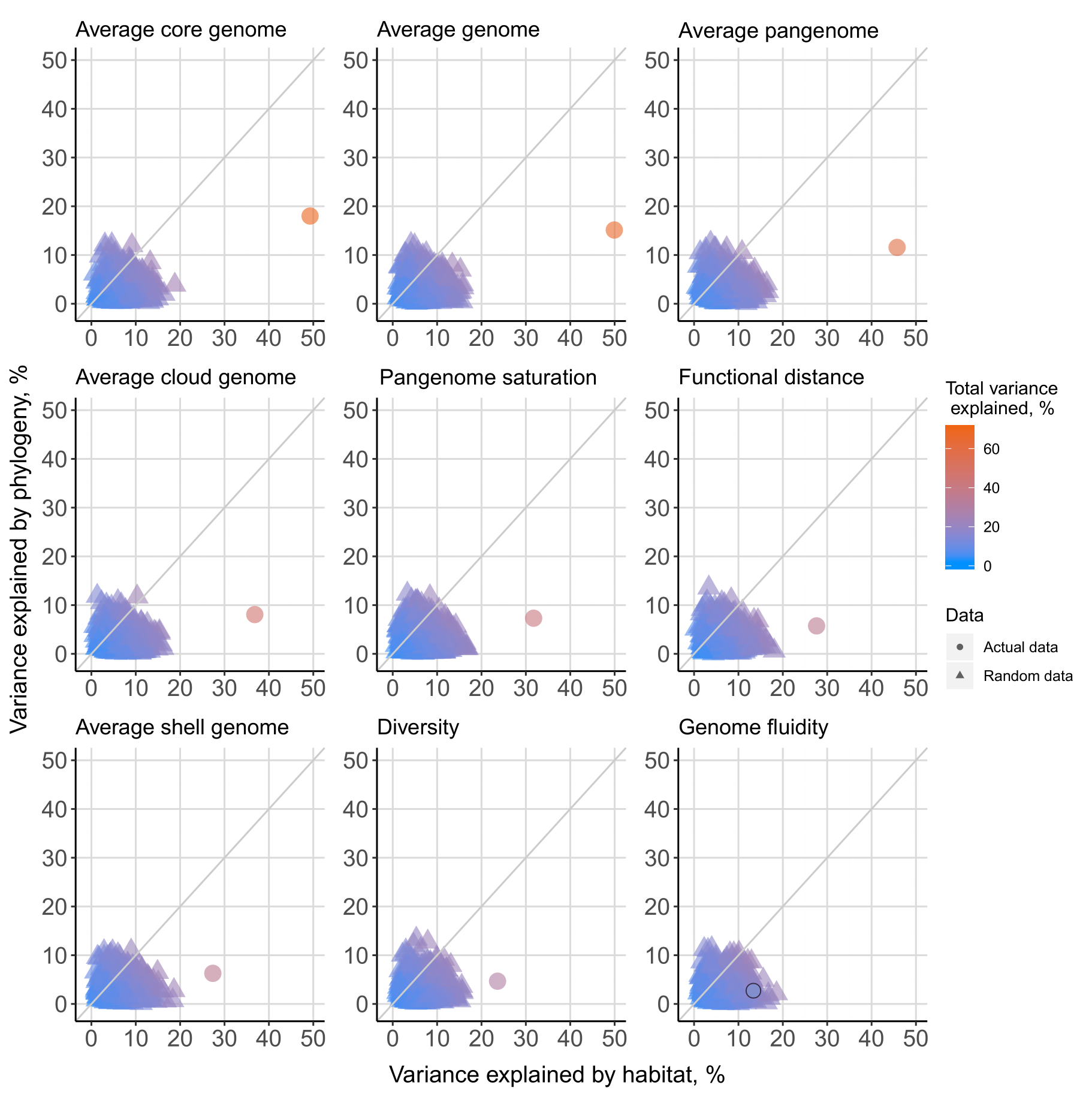


**Supplementary Figure 6**. Randomized phylogenetic and habitat PCs explain a smaller fraction of the variance than actual data for the core genome size and the average genome.
